# Uncovering essential lncRNAs through transcriptome-scale CRISPR-Cas13 screening

**DOI:** 10.1007/s44307-025-00082-8

**Published:** 2025-09-15

**Authors:** Jiahua Si, Xinming Su, Zhuoyan Jin, Shiwei Duan

**Affiliations:** Department of Clinical Medicine, School of Medicine, Hangzhou City University, Hangzhou, Zhejiang China

**Keywords:** CRISPR, Cas13, RNA targeting, LncRNA, Transcriptome

## Abstract

Approximately 75% of the human genome is transcribed into RNA, yet less than 5% encodes proteins, with the majority producing non-coding RNAs (ncRNAs). Among them, long non-coding RNAs (lncRNAs) represent a major class that exerts broad regulatory influence across cellular processes, disease contexts, and developmental stages. Despite their potential as biomarkers and therapeutic targets, their low sequence conservation, limited abundance, and structural complexity present significant challenges for functional characterization. Traditional RNA interference and CRISPR-Cas9–based methods have offered partial insights but remain limited in efficiency, specificity, and scalability. To address these barriers, Neville E. Sanjana’s team developed CaRPool-seq, a transcriptome-scale CRISPR-Cas13 screening platform that directly targets RNA. Applying this approach across diverse human cell lines, they identified 778 essential lncRNAs, including 46 universally required for survival, with distinctive structural features and functional independence from neighboring protein-coding genes. Integration with single-cell transcriptomics revealed their critical roles in cell-cycle regulation, apoptosis, and developmental gene expression, as well as aberrant expression patterns in cancer linked to patient outcomes. This study establishes CRISPR-Cas13 as a precise and scalable strategy for lncRNA functional discovery, expanding opportunities for biomarker identification, therapeutic development, and precision medicine.

## Main Text

Approximately 75% of the human genome is transcribed into various RNAs, yet less than 5% of these sequences encode proteins. The majority of genomic DNA is transcribed into non-coding RNAs (ncRNAs) Chen and Kim 2024. ncRNAs represent a highly complex family of molecules that are deeply integrated into regulatory networks and play crucial roles in diverse cellular processes. Their expression is disease-, tissue-, and cell-type-specific, making them attractive candidates for targeted therapies and personalized medicine Chen and Kim 2024. 

Among ncRNAs, long non-coding RNAs (lncRNAs) constitute a major class, defined as transcripts longer than 200 nucleotides, and account for 80%–90% of all ncRNAs Statello et al., 2021. Although lncRNAs generally lack protein-coding capacity due to the absence of a valid open reading frame, their splicing processes resemble those of messenger RNAs (mRNAs) Statello et al., 2021. Compared with other ncRNAs, lncRNAs exhibit greater diversity in biogenesis, molecular function, and cellular impact, which has made them a central focus of both basic and translational research Statello et al., 2021. Their expression specificity provides opportunities for precise therapeutic targeting and prognostic evaluation; however, their relatively low sequence conservation and expression abundance compared with mRNAs pose challenges for detection, quantification, and functional characterization in disease contexts Coan et al., 2024.

Functional studies of lncRNAs currently rely heavily on multi-omics approaches (such as RNA sequencing and transcriptome microarrays) as well as perturbation-based strategies for target discovery Coan et al., 2024. Among these, direct perturbation of lncRNA expression or activity remains the most informative, as it enables a clear evaluation of their roles in cellular phenotypes Coan et al., 2024. RNA interference (RNAi) was once widely used for such purposes, but due to high costs, annotation issues, and limited scalability, it has been applied only in a small number of systematic screens Coan et al., 2024. More recently, CRISPR-based technologies—including CRISPR knockout, CRISPR interference (CRISPRi), and CRISPR activation (CRISPRa)—have been adopted for lncRNA screening, filling critical gaps in the field. These methods enable transient, reversible, and direct regulation of lncRNA expression, allowing efficient high-throughput functional identification Coan et al., 2024. However, the DNA-targeting nature of CRISPR-Cas9 limits its ability to effectively disrupt lncRNAs with a single guide RNA. Complete knockout often requires dual sgRNAs to induce large genomic deletions or the generation of homozygous knockout cell lines via monoclonal screening. This process is inefficient, prone to off-target effects, and may inadvertently disrupt adjacent or overlapping protein-coding genes Coan et al., 2024.

To overcome these challenges, Neville E. Sanjana’s team developed CaRPool-seq (Cas13 RNA Perturb-seq), a transcriptome-scale CRISPR screening platform based on CRISPR-Cas13 Liang et al., 2024. Unlike Cas9, Cas13 targets RNA directly, enabling specific guide RNAs (gRNAs) to bind and silence lncRNAs at the transcript level. This approach minimizes nonspecific DNA editing, reduces off-target interference, and enhances the precision of functional ncRNA discovery.

**Fig. 1:**
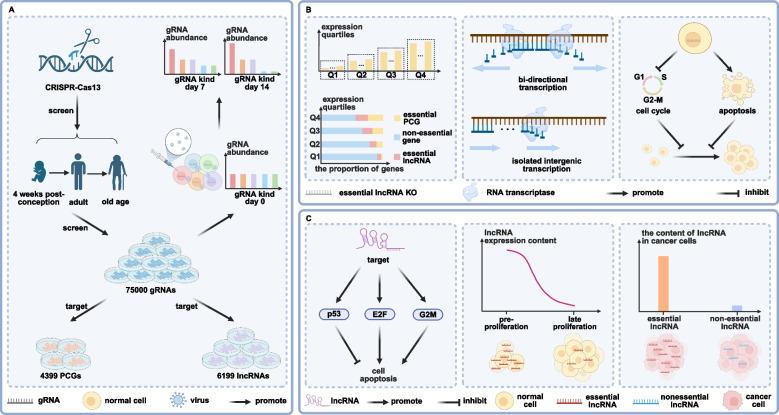
Functional Characterization of Essential lncRNAs through CRISPR-Based Screening **A** Gene Library Construction and Screening. Using CRISPR technology, the authors constructed a comprehensive gene library reflecting human physiological development from early embryogenesis (4 weeks) to old age. This library comprised 75,000 gRNAs targeting 4,399 protein-coding genes (PCGs) and 6,199 long non-coding RNAs (lncRNAs). Lentiviral delivery of the gRNAs into five human cell lines enabled functional screening, with gRNA abundance measured at days 0, 7, and 14. A significant reduction in gRNA abundance was observed for those targeting lncRNAs with essential cellular functions, indicating selective pressure against their depletion. **B** Comparison of Essential lncRNAs and PCGs. Analysis revealed that both essential PCGs and essential lncRNAs were enriched among the most strongly depleted gRNAs. Unlike PCGs, essential lncRNAs tended to act at relatively low expression levels. Structurally, the majority of essential lncRNAs were identified as bidirectional transcripts, with only a minority classified as isolated intergenic transcripts. Functionally, these lncRNAs were shown to regulate cell proliferation primarily by inducing apoptosis and impairing cell-cycle progression. **C **lncRNA Expression Dynamics and Regulatory Mechanisms. Essential lncRNAs exerted their effects on apoptosis and cell-cycle regulation through key signaling pathways, including p53, E2F, and G2M. Their expression was significantly elevated during the early stages of cell proliferation and declined at later stages. Notably, in cancer cells, essential lncRNAs exhibited greater expression variability compared with non-essential lncRNAs, underscoring their potential roles in tumor heterogeneity and disease progression

As illustrated in Fig. 1A, the researchers used lncRNA expression data from seven human organs across 26 developmental stages (from 4 weeks gestation to late adulthood) to design a CaRPool-seq gRNA library targeting 6,199 lncRNAs and 4,390 protein-coding genes (PCGs). Lentiviral delivery of the library into five human cell lines (HAP1, HEK293T, K562, MDA-MB-231, and THP1) enabled systematic assessment of the impact of lncRNA depletion on cell survival. The large-scale screen identified 778 lncRNAs essential for survival in at least one cell line, of which 46—including MALAT1 and MIR17HG—were essential across all five. These universally essential lncRNAs displayed high expression levels, strong conservation of function, and distinctive structural features, being predominantly antisense or bidirectional transcripts rather than intergenic ones. Interestingly, most essential lncRNAs did not exhibit significant co-expression with neighboring PCGs, suggesting their functional independence and potential roles in long-range or cross-regulatory mechanisms.

To further explore the spatiotemporal roles of lncRNAs, the team integrated CaRPool-seq with single-cell transcriptome analysis (Fig. 1B). This revealed that depletion of essential lncRNAs impaired cell-cycle progression and promoted apoptosis. Gene set enrichment analysis (GSEA) indicated strong associations with pathways governing proliferation, including MYC, mTOR, and p53, independent of PCG regulation. Many essential lncRNAs also exhibited dynamic developmental expression patterns (Fig. 1C): highly expressed during early embryogenesis, then downregulated over time, with notable enrichment in proliferative tissues such as brain, heart, liver, and kidney. Analysis of ~ 9,000 tumor transcriptomes further revealed aberrant lncRNA expression signatures in cancers, co-expression with oncogenic drivers, and correlations with patient survival outcomes.

This study marks a significant advance in lncRNA biology by demonstrating that transcriptome-scale, RNA-targeting CRISPR-Cas13 screens can identify essential lncRNAs with high precision. Beyond lncRNAs, this approach can be extended to other ncRNAs, including microRNAs and circular RNAs. Nonetheless, challenges remain: the structural complexity of lncRNAs complicates gRNA design; current libraries lack full transcriptome coverage; and downstream validation in disease-relevant models is still needed. Future progress will depend on more sophisticated bioinformatics tools—potentially integrating artificial intelligence with RNA structural modeling—to optimize gRNA design, reduce off-target effects, and enhance scalability.

In summary, this study reveals the indispensable roles of lncRNAs in cellular survival and development, highlights their disease relevance, and provides a powerful RNA-focused CRISPR-Cas13 platform for functional genomics. With continued improvements in screening technologies and expanded clinical validation, these advances may accelerate the translation of lncRNA research into precision diagnostics and therapies.

## Data Availability

Not applicable.

## Abbreviations

CRISPR: Clustered regularly interspaced short palindromic repeats
CasRPool-seq: Cas13 RNA Perturb-seq
gRNAs: Guide RNAs
LncRNAs: Long noncoding RNAs
mRNA: Messenger RNA
ncRNA: Non-coding RNA
PCGs: Protein-coding genes
